# Data-Driven User Feedback: An Improved Neurofeedback Strategy considering the Interindividual Variability of EEG Features

**DOI:** 10.1155/2016/3939815

**Published:** 2016-08-18

**Authors:** Chang-Hee Han, Jeong-Hwan Lim, Jun-Hak Lee, Kangsan Kim, Chang-Hwan Im

**Affiliations:** Department of Biomedical Engineering, Hanyang University, Seoul 133-731, Republic of Korea

## Abstract

It has frequently been reported that some users of conventional neurofeedback systems can experience only a small portion of the total feedback range due to the large interindividual variability of EEG features. In this study, we proposed a data-driven neurofeedback strategy considering the individual variability of electroencephalography (EEG) features to permit users of the neurofeedback system to experience a wider range of auditory or visual feedback without a customization process. The main idea of the proposed strategy is to adjust the ranges of each feedback level using the density in the offline EEG database acquired from a group of individuals. Twenty-two healthy subjects participated in offline experiments to construct an EEG database, and five subjects participated in online experiments to validate the performance of the proposed data-driven user feedback strategy. Using the optimized bin sizes, the number of feedback levels that each individual experienced was significantly increased to 139% and 144% of the original results with uniform bin sizes in the offline and online experiments, respectively. Our results demonstrated that the use of our data-driven neurofeedback strategy could effectively increase the overall range of feedback levels that each individual experienced during neurofeedback training.

## 1. Introduction

Neurofeedback is a type of biofeedback technology that has generally been used to train the ability of self-regulation based on the real-time analysis of neural signals such as electroencephalography (EEG), magnetoencephalography (MEG), and real-time functional magnetic resonance imaging (fMRI) [1]. Over the past decades, several experimental studies have demonstrated that neurofeedback training can be used effectively for the treatment of patients with various psychiatric diseases or neurological disorders such as attention deficit hyperactivity disorder (ADHD), autism, depression, Tourette syndrome, insomnia, and epilepsy [2–7]. Furthermore, recent studies have reported that neurofeedback training can temporally enhance the cognitive performances of healthy individuals [8–10]. Thanks to these positive effects, neurofeedback has been gaining increased attention [11].

Among the various neural signal acquisition modalities including EEG, MEG, and fMRI [12–14], EEG has been the most widely used for implementing neurofeedback systems due to its several advantages over other neuroimaging modalities, such as high temporal resolution, portability, and reasonable cost [15]. Since the 1960s, when the concept of EEG-based neurofeedback was first introduced [16], most of the EEG-based neurofeedback studies used a spectral power of a specific frequency band [17]. For example, “alpha” neurofeedback training is known to improve cognitive performance in human subjects [8], and “beta” neurofeedback training is known to affect attentional processing [18]. Recent studies have adopted a variety of EEG features, such as the ratio of two or more spectral powers, to improve the overall performance of neurofeedback [19].

Despite the recent development of EEG-based neurofeedback strategies, however, the EEG-based neurofeedback systems still suffer from some limitations. One of the most representative limitations is the large interindividual variability of the EEG features that have been used for neurofeedback [20–22]. Due to the large variability of individual EEG signals, it is generally difficult to develop “universal” neurofeedback systems that can be applied to all users without any time-consuming customization or individualization processes [23–25]. Therefore, some users of the current neurofeedback systems may experience only a small portion of the entire feedback range (e.g., see Figure 3 in advance). Although the use of a customization or individualization session before using a neurofeedback system would enhance the performance of the neurofeedback systems, such time-consuming and cumbersome sessions might decrease the satisfaction of the users. Therefore, it is still necessary to make an effort to develop EEG features with small interindividual variations or to increase the feedback ranges that each individual experiences.

In the present study, we attempted to make the users of the neurofeedback systems experience a wider range of auditory or visual feedback. To this aim, we proposed a new neurofeedback strategy, named as the data-driven user feedback strategy, which uses nonuniform bin sizes to divide the entire range of an EEG feature into many bins, each of which is assigned to a corresponding feedback level, based on the offline EEG database acquired from a group of individuals.

## 2. Methods

### 2.1. Subjects

Two groups of healthy subjects were enrolled in our experiments. The first group, consisting of 22 healthy subjects (17 males and 5 females; mean age 23.73 ± 3.12 years), participated in offline experiments to construct an EEG database, and, the second group, consisting of five healthy subjects (four males and one female; mean age 25.20 ± 1.17 years), participated in online experiments to validate the performance of the proposed neurofeedback strategy. Subjects who participated in the offline experiment were not enrolled in the online experiment again because we wanted to test whether the new subjects could show good performance using the proposed neurofeedback strategy based on the other participants' database. All participants had normal or corrected-to-normal vision and none had a previous history of neurological, psychiatric, or other severe diseases that might otherwise affect the experimental results. Before each experiment, comprehensive information on the experiments was given to each participant, and written informed consent was obtained from each subject. This study was reviewed and approved by the Institutional Review Board Committee of Hanyang University.

### 2.2. Experimental Procedure

**Figure 1:**
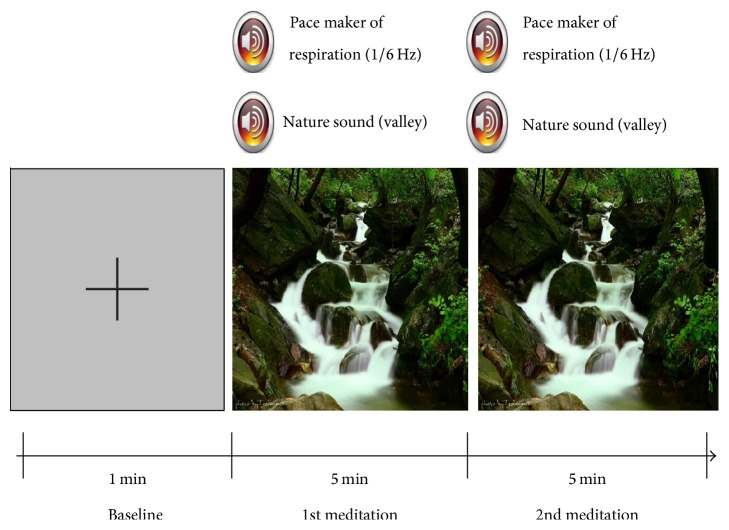
A schematic diagram describing our experimental paradigm. A babbling brook sound, a picture of a beautiful valley, and a quiet pure-tone beep sound (respiration pacemaker) with a period of three seconds were simultaneously provided to each study participant.

To verify the feasibility of the proposed neurofeedback strategy, we conducted offline and online experiments. In the offline experiment, each participant in the first group performed a “meditation” paradigm, which helped the study participants to relax. The meditation paradigm consisted of resting, first meditation, and second meditation periods (Figure 1). During the resting period, a black fixation cross appeared at the center of an LCD monitor for 1 min, while each subject was asked to gaze at the fixation cross without moving his or her body. Then, a babbling brook sound, a picture of a beautiful valley, and a quiet pure-tone beep sound with a period of three seconds were simultaneously provided to each subject. The study participants were asked to take a slow breath to the beat of the beep sound, while they consistently watched the picture on the monitor and listened to the brook sound. This five-minute session was repeated twice with a short break time. In the online experiments, each participant in the second group performed the same meditation task as in the offline experiment, except that real-time visual feedback was provided reflecting the participant's current meditation state. The size of a circle and the length of a bar displayed on a monitor varied according to the level of the participant's meditation state.

### 2.3. EEG Recording and Preprocessing

EEG signals were recorded using a multichannel EEG acquisition system (ActiveTwo, BioSemi, Amsterdam, Netherlands). Two EEG electrodes (Fp1 and Fp2) were mounted on the prefrontal area of the subject's scalp according to the international 10–20 system, assuming a headband-type portable EEG neurofeedback system. The ground electrode was replaced with two electrodes, a common mode sense (CMS) active electrode and a driven right leg (DRL) passive electrode, both of which were located in the posterior region. The EEG data were sampled at 2,048 Hz, and then the spectral power of the alpha band (8–12 Hz) was calculated using a fast Fourier transform.

### 2.4. Data-Driven Neurofeedback Strategy

As aforementioned, neurofeedback can be used to enhance cognitive performance of an individual or treat patients with neuropsychiatric diseases and neurological disorders. Although there is no formal procedure for the conventional neurofeedback training, the entire dynamic range of an EEG feature is usually evenly divided into multiple segments, each of which is then assigned to a corresponding level of auditory or visual feedbacks. In this case, some users who have narrow dynamic ranges of the EEG feature might accordingly experience relatively narrow ranges of feedback compared with other users who have broad dynamic ranges. The dynamic range of the EEG feature of an individual can be recorded before the neurofeedback training and set differently for each individual, which is referred to as the customization session; however, this customization session generally needs to be conducted every time a user wants to try the neurofeedback system because of the high intertrial variability of EEG data [26, 27]. In the present study, to enable most users to experience a wider dynamic range of feedback during neurofeedback training without a customization process, we used nonuniform bin sizes to divide up the entire EEG feature range. The size of each bin was determined based on the grand distribution of the EEG feature (in this study, alpha band power averaged over Fp1 and Fp2 was used to evaluate meditation states). The fundamental concept of the proposed method was introduced in our preliminary study [28]. This paper is the extended version of the preliminary study, and we additionally confirmed its online performance using other EEG dataset in this study. The proposed method will be explained in detail in the next paragraph.

**Figure 2:**
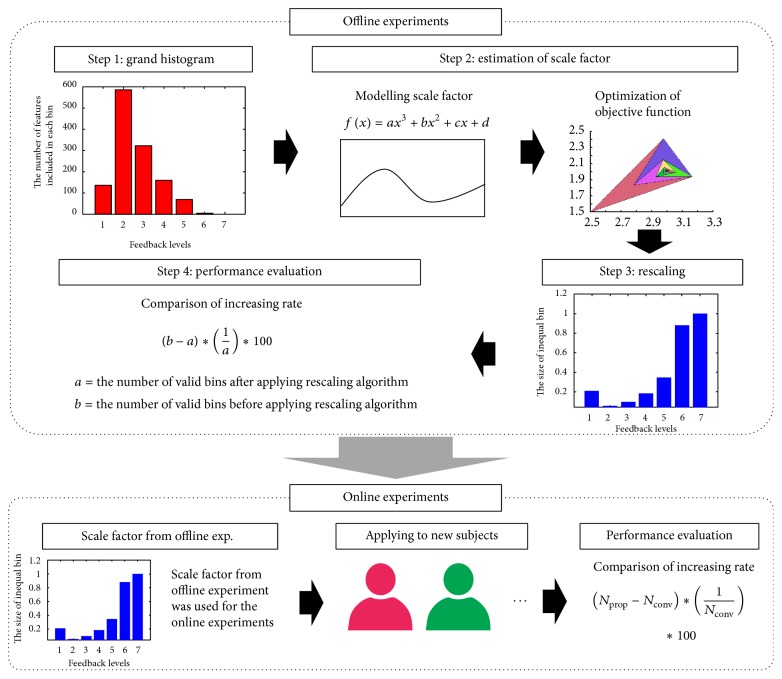
A schematic illustration of the overall procedure of the proposed neurofeedback strategy.

In the first step, a database of an EEG feature was constructed, while a group of participants was performing a specific mental task (a meditation task in this study). Using the database, a grand histogram was obtained. The grand histogram could show overall distribution of the EEG feature values. In this study, we used frontal alpha band (8–12 Hz) power as the EEG feature [29–31]. Alpha band powers were evaluated for 60 EEG epochs with a length of 2 seconds. Each epoch was randomly sampled from artifact-free periods of resting EEG (20 epochs) as well as EEG recorded during the first- and second-meditation tasks (20 epochs each). The initial bin size of the histogram was set arbitrarily; we first divided the entire dynamic range into seven bins (please see step 1 of Figure 2), where each bin represents evenly divided frequency bands. In the second step, a scale factor was introduced, which determined the number of subdivisions of each initial bin. Basically, when the number of EEG epochs included in an initial bin range is large, the bin is divided into relatively more subdivisions. For example, if the scale factor of an initial bin is 0.1, the size of the subdivisions of the bin becomes 0.1 times the original bin size; that is, the bin is divided into 10 subdivisions. The scale factor was modeled with a third-order polynomial, *f*(*x*) = *ax* ^3^ + *bx* ^2^ + *cx* + *d*, where *x* is the number of EEG epochs included in an initial bin range and coefficients *a*, *b*, *c*, and *d* are unknowns that need to be determined. The order of the polynomial was selected empirically after confirming that the third-order polynomial could model the overall shape of the scale factor fairly well. We also limited the maximum of the scale factor to 50 to avoid an excessive increment of the number of subdivisions. The coefficients of the polynomial were determined using an optimization procedure with an objective function defined as the average increment in the number of feedback levels that each individual experiences. More specifically, the following equation was used as the objective function of the optimization:

(1) Increasing  Rate%=Nprop−Nconv×1Nconv×100,

where *N*_conv_ and *N*_prop_ represent the average numbers of feedback levels that each individual experiences when conventional and proposed neurofeedback strategies are used, respectively. In calculating the objective function, the total number of feedback levels of the conventional neurofeedback strategy, *N*_conv_, was set to be always identical to that of the proposed neurofeedback strategy, *N*_prop_. Contrary to the proposed strategy, the entire range of the EEG feature was evenly divided in the case of the conventional neurofeedback strategy. A Nelder-Mead simplex direct search algorithm implemented in a MATLAB optimization toolbox (MathWorks, Natick, MA, USA) was used for the optimization. The algorithm was designed to solve classical unconstrained optimization problems of minimizing a given nonlinear function without a need for the calculation of derivatives [32]. We selected this algorithm because it is simple to use and could quickly yield reliable results. In the final step, each initial bin was divided into smaller subdivisions based on the optimized scale factor, and, accordingly, the total number of feedback levels was also adjusted.

## 3. Results

To evaluate whether study participants performed the given meditation task well, a statistical analysis was performed. A paired *t*-test was applied to confirm a statistically significant difference in the alpha band powers in the resting and meditative states. The result of the statistical analysis showed that the alpha power was significantly increased during meditation periods (2.19 ± 0.69) compared to resting periods (1.88 ± 0.76) (units: *μ*V^2^; *p* value: 0.03). This result is in line with the findings in many previous studies that consistently reported increased frontal alpha band activity during meditation tasks [29–31].

**Figure 3:**
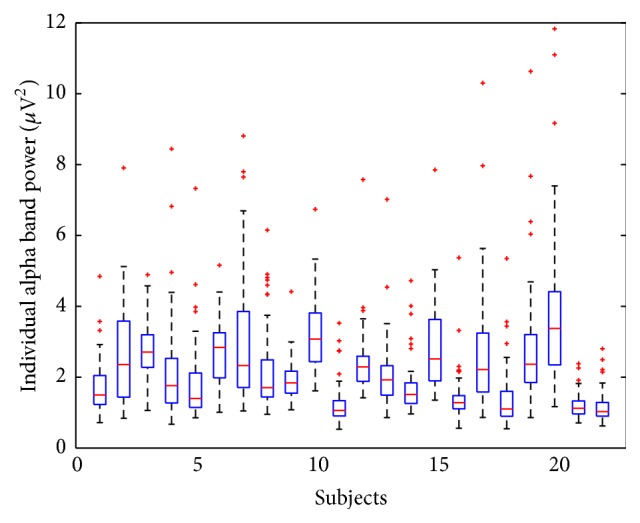
Changes in the EEG meditation feature of each individual participant acquired during resting and meditation periods.

Figure 3 shows the distributions of the frontal alpha powers of each participant, which were recorded during both resting and meditation conditions. As seen in the figure, large interindividual variability was observed. Specifically, some participants (subject numbers 11, 16, 21, and 22) showed relatively small dynamic ranges of the alpha powers, while other participants (subject numbers 2, 7, 10, 15, 17, and 20) showed relatively large dynamic ranges.

**Figure 4:**
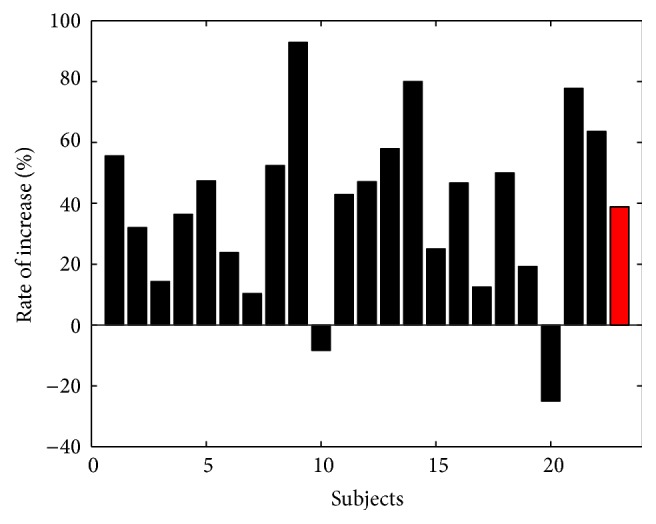
Results of offline experiments. The number of feedback levels that each subject experienced was increased in most participants. A red bar shows the average rate of increase.

Figure 4 shows the increasing rate of each individual participant's feedback levels after the optimized scale factor was applied. The average number of feedback levels that each individual experienced increased to 139% of the original results with uniform bin sizes when the proposed neurofeedback strategy was applied. Twenty participants experienced increased numbers of feedback levels, while only two participants experienced reduced numbers of feedback levels, and the rate of decrement for those subjects was only about 20%.

**Figure 5:**
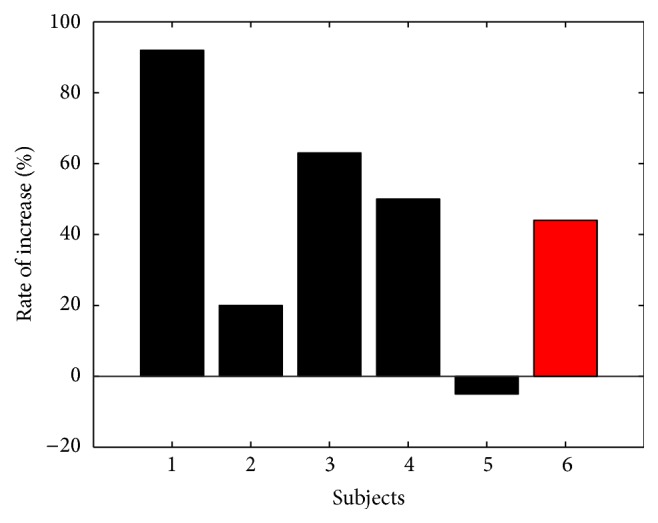
Results of online experiments. The average number of feedback levels that each participant experienced increased to 144% of the original results with uniform bin size. A red bar shows the average rate of increase.

Figure 5 shows the results of the online experiments for validation. In the online experiments with real-time feedback, the scale factor optimized using the offline datasets was directly applied without modification. When the proposed neurofeedback strategy was used, the average number of feedback levels that each participant experienced increased to 144% of the original results with uniform bin sizes. Four out of five participants experienced increased numbers of feedback levels, while one participant experienced a reduced number of feedback levels, with a decrement of less than 5%.

To demonstrate further the practicality of the proposed neurofeedback strategy, we asked participants to stare continuously at the real-time feedback (a varying circle and a varying vertical bar) as well as the picture of a valley during the online experiment. The size of the circle and the length of the bar varied with respect to the changes in the EEG meditation feature, but the feedback levels were set differently according to the conditions (conventional or proposed neurofeedback strategy). The supplementary movie file demonstrates that the user of the neurofeedback system could experience wider ranges of feedback without any training sessions or predata acquisition sessions (see supplementary movie file in Supplementary Material available online at http://dx.doi.org/10.1155/2016/3939815).

## 4. Discussion

It has been frequently reported in the literature and also shown in this study that some neurofeedback users can experience only a small portion of the total feedback range due to the large interindividual variability of EEG features. Most previous EEG-based neurofeedback studies focused on developing an individual customization strategy with the aim of addressing the large interindividual variability issue [23–25]. However, the customization strategy still has several difficulties, because it necessarily requires time-consuming and cumbersome calibration sessions before the neurofeedback training. Even after an individual customization session, the dynamic range of the EEG features of an individual can vary day by day, and, thus, repetitive training sessions are often required. In our present study, to enable most users to experience a wider range of feedback levels without any customization processes, an improved neurofeedback strategy was proposed. In contrast to the general neurofeedback strategy that uses a uniform bin size, the proposed neurofeedback strategy used nonuniform bin sizes to divide the entire range of EEG features based on the EEG database recorded from a group of individuals. The number of subdivisions in each bin was determined through an optimization process using a simplex search algorithm with an objective function to maximize the average number of feedback levels that each individual experienced. In this study, the EEG feature database was constructed using EEG data recorded from 22 healthy participants, while they were performing a meditation task paradigm. Then, the performance of the proposed neurofeedback strategy was confirmed through online experiments with five additional participants. The results of the proposed neurofeedback strategy exhibited increments in the numbers of feedback levels as high as 139% and 144% of the original results with a uniform bin size for the offline and online experiments, respectively.

Although the proposed neurofeedback strategy might not be the ultimate solution to circumvent the general limitations of the current neurofeedback approaches, that is, the large interindividual variability issue, this strategy can enhance the performance of the neurofeedback systems without the need for customization or individualization sessions. If the optimized scale factor to adjust the sizes of nonuniform bins is predetermined using a large EEG database, most new users can use the neurofeedback training programs directly and experience increased feedback ranges. In this study, we only used a meditation task paradigm that can be used to train users to stay in a relaxed state; however, our neurofeedback strategy can also be generally used for other neurofeedback applications such as the treatment of patients with ADHD or depression. In other words, the proposed strategy can be used with various EEG features such as frontal alpha asymmetry and coherence between different channels if prerecorded databases are available. Notably, our strategy showed fairly good performance despite the fact that only a small number of EEG datasets were used to construct the feature database. In our present study, the database was constructed using EEG data recorded from only 22 subjects, but it showed an increment of feedback levels of around 50% in the online experiments. We expect that the performance of the proposed neurofeedback strategy would be further enhanced if a larger database could be used.

**Figure 6:**
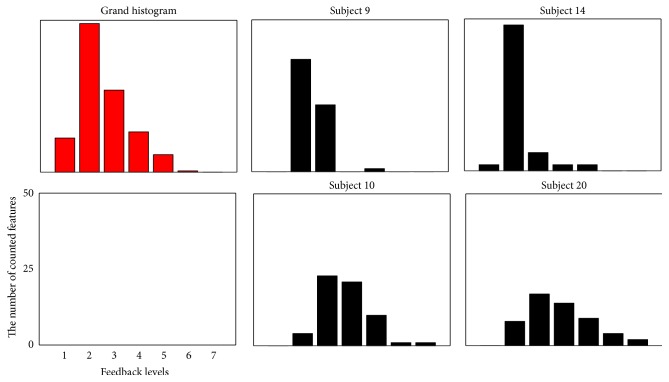
Grand and individual histograms of EEG meditation features. The red graph (upper left) shows the grand histogram, and the black graphs (the other four histograms) indicate the histograms of four individual participants.

In both the offline and online experiments, few study participants (subjects 10 and 20 in the offline experiments; subject 5 in the online experiments) experienced reduced numbers of feedback levels after applying the proposed neurofeedback strategy (see Figures 4 and 5). We found that the participants who did not show good performance had histogram distributions significantly different from that of the grand histogram. In Figure 6, two participants, subjects 9 and 14, showed distributions similar to that of the grand histogram and thus showed the best and second-best increments in the numbers of feedback levels. In contrast, the other two participants included in Figure 6 (subjects 10 and 20, who showed the worst performances) showed distributions considerably different from that of the grand histogram. Nevertheless, about 90% of all study participants showed enhanced performance, and, thus, the proposed neurofeedback strategy is expected to be effective for most users. We expect that these exceptional cases can be potentially reduced if a larger EEG database is used and a better modeling of the scale factor is possible, which is a topic we would like to pursuit in future studies. In addition, further experiments need to be conducted in future studies in order to investigate the test-retest reliability of our method, considering the high intertrial variability of EEG features.

## Supplementary Material

The supplementary movie file demonstrates that the user of the neurofeedback system could experience wider ranges of feedback without any trainning sessions or pre-data acquisition sessions.
